# Restoration of radiation therapy-induced salivary gland dysfunction in mice by post therapy IGF-1 administration

**DOI:** 10.1186/1471-2407-10-417

**Published:** 2010-08-10

**Authors:** Oliver Grundmann, Jamia L Fillinger, Kerton R Victory, Randy Burd, Kirsten H Limesand

**Affiliations:** 1 Department of Nutritional Sciences, University of Arizona, Tucson, AZ, USA

## Abstract

**Background:**

Radiotherapy for head and neck cancer results in severe and chronic salivary gland dysfunction in most individuals. This results in significant side effects including xerostomia, dysphagia, and malnutrition which are linked to significant reductions in patients' quality of life. Currently there are few xerostomia treatment approaches that provide long-term results without significant side effects. To address this problem we investigated the potential for post-therapeutic IGF-1 to reverse radiation-induced salivary gland dysfunction.

**Methods:**

FVB mice were treated with targeted head and neck radiation and significant reductions in salivary function were confirmed 3 days after treatment. On days 4-8 after radiation, one group of mice was injected intravenously with IGF-1 while a second group served as a vehicle control. Stimulated salivary flow rates were evaluated on days 30, 60, and 90 and histological analysis was performed on days 9, 30, 60, and 90.

**Results:**

Irradiated animals receiving vehicle injections have 40-50% reductions in stimulated salivary flow rates throughout the entire time course. Mice receiving injections of IGF-1 have improved stimulated salivary flow rates 30 days after treatment. By days 60-90, IGF-1 injected mice have restored salivary flow rates to unirradiated control mice levels. Parotid tissue sections were stained for amylase as an indicator of functioning acinar cells and significant reductions in total amylase area are detected in irradiated animals compared to unirradiated groups on all days. Post-therapeutic injections of IGF-1 results in increased amylase-positive acinar cell area and improved amylase secretion. Irradiated mice receiving IGF-1 show similar proliferation indices as untreated mice suggesting a return to tissue homeostasis.

**Conclusions:**

Post-therapeutic IGF-1 treatment restores salivary gland function potentially through normalization of cell proliferation and improved expression of amylase. These findings could aid in the rational design of therapy protocols or drugs for the treatment of radiation-induced salivary gland dysfunction in patients who have completed their anti-cancer therapies.

## Background

The treatment of head and neck cancer commonly involves fractionated radiation therapy which results in significant side effects including xerostomia, dysphagia, and increased infection rates in most patients [1-3]. Although attempts are made to limit radiation exposure to spare normal tissues, the close proximity of the salivary glands to the treatment field leads to impairment of physiological function and secondary side effects that impact quality of life for patients [4]. A reduction of saliva flow rates by 50-60% with accompanying changes in saliva composition ensues during the first week of continued radiation therapy which has been associated with a loss of acinar cells and glandular shrinkage [5,6]. Serous acinar cells of the parotid gland are the main contributor of protein and water to the composition of saliva [7,8]. It has been shown in humans that radiation-induced defects in amylase production by the parotid acinar cells lead to significant decreases in amylase concentration in stimulated saliva [9]. These immediate pathophysiological and structural changes may contribute to the development of chronic symptoms such as malnutrition, mucositis, and permanent reduction in saliva flow rates based on acinar cell attrition and replacement with fibrotic tissue [10-12].

Current treatment approaches focus mainly on preventing radiation-induced xerostomia. The only preventative therapy currently available is amifostine which is linked to unfavorable side effects such as hypotension, vomiting, and allergic reaction with a high discontinuation rate [13]. A promising drug for xerostomia prevention is tempol, which is currently being evaluated in clinical trials [14]. Since reductions in salivary flow rates are believed to be the cause for many of the secondary complications, palliative therapy with cholinergic agonists such as pilocarpine and cevimeline have been proposed to stimulate salivary flow [15,16]. However, these treatments have transient efficacy and limited success due to side effects. Promising research has been conducted in recent years that have the potential to affect the development of new treatment approaches for salivary gland dysfunction including growth factors, gene transfer, artificial salivary glands, and stem cell transplantation (reviewed in [17]).

A number of animal models have been utilized in order to understand the sensitivity of the salivary glands to therapeutic radiation (reviewed in [17]). Consistent with the human studies, all of these models demonstrate the acute and chronic loss of function; however the mechanisms responsible for the alterations in glandular physiology are debated. The mechanism of loss of acinar cell area seems to involve an apoptotic process which appears to be mediated through a p53-dependent pathway [18,19]. Transgenic mice expressing a constitutively activated mutant of Akt1 (myr-Akt1) suppressed radiation-induced apoptosis *in vitro *and *in vivo *by regulating the activation of p53 [18]. The use of intravenous injection of recombinant IGF-1 before radiation exposure has been shown to activate endogenous Akt in the salivary glands and suppress radiation-induced apoptosis [16]. Importantly, the suppression of apoptosis correlated with improved salivary function following radiation treatment.

Levels of radiation-induced apoptosis in mouse models appear to be dose dependent; however, the reductions in salivary flow rate are similar regardless of radiation dose [16,18-20]. Salivary glands present with significantly elevated levels of apoptotic cells within the first 24 hours after exposure to a single dose of ionizing radiation of which the majority are acinar cells [16,18-20]. In animal models of radiation-induced damage, reductions in salivary flow rates have been reported within 3 days post-treatment and persist out to one year (reviewed in [17]). In contrast to clinical studies, little data is available regarding the recovery from radiation-induced salivary gland dysfunction in animal models. Because apoptosis has been reported to occur initially following radiation in parotid salivary glands, the rate of acinar cell proliferation may provide further insight into restoration of radiation-induced tissue damage that leads to normal salivary flow rates in mice treated with post therapy IGF-1. The current study investigates the relationship between a single dose radiation exposure (5 Gy) and the ability of post radiation therapy administration of recombinant IGF-1 to restore salivary gland function.

## Methods

### Mice

Female FVB mice between 4-5 weeks old, obtained from Taconic (Taconic, Hudson, NY) were used for all experiments. Animals were maintained on a 12 h light/dark cycle in accordance with the protocols approved by the University of Arizona Institutional Animal Care and Use Committee (IACUC).

### Radiation Treatment

**Figure 1:**
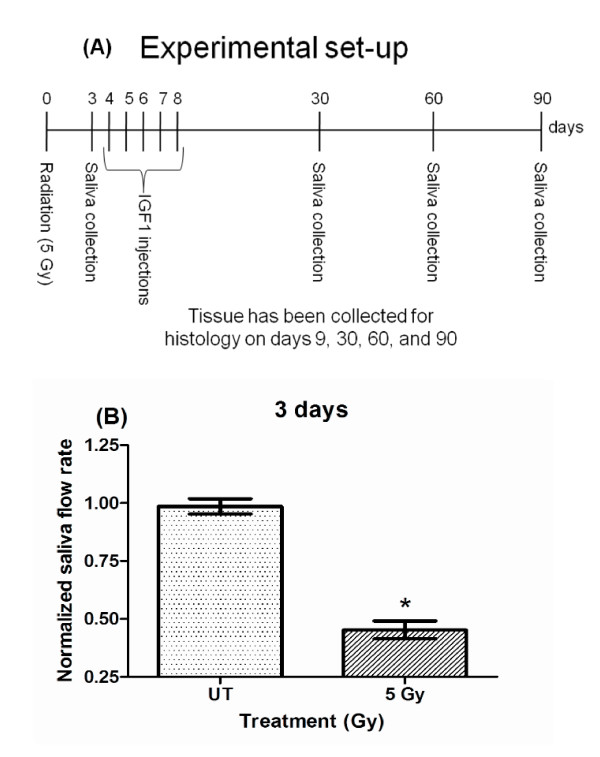
**Experimental setting (A) and reduction in salivary flow rates on day 3 following radiation (B)**. The head and neck region of FVB mice was exposed to a single dose of 5 Gy radiation. Stimulated salivary flow rates were determined as described in the materials and methods section on day 3 with 81 animals in the untreated (UT) and 72 animals in the treated group. Significant differences (p < 0.05) were determined using an ANOVA followed by a Bonferroni test. (*) in figure 1B over 5Gy treatment group indicates significant differences between groups.

Mice were anesthetized intraperitoneally with avertin (tribromoethanol) in a dose of 0.4 to 0.6 mg/kg prior to radiation treatment. Mice were then irradiated with a single fraction of radiation (5 Gy) to the head and neck region (Cobalt-60 Teletherapy unit from Atomic Energy of Canada Ltd Theratron-80) as previously described (day 0; figure 1A) [19]. This results in irradiation of the submandibular, sublingual, parotid, and minor salivary glands. The 5 Gy radiation dose was chosen based on our previous work demonstrating the dose caused elevated levels of p53 protein, activation of apoptosis, absence of cell cycle arrest, and loss of salivary function [18,19,21,22]. The rest of the body (below clavicle) was shielded with >6 mm thick lead which blocked ~75% of the radiation to avoid systemic effects of radiation. Radiation dosimetry calculations and maintenance of the cobalt source are conducted by the Experimental Radiation Shared Service of the Arizona Cancer Center. Day 3 stimulated salivary flow rates were used to confirm significant reductions due to radiation exposure (figure 1B) and then all animals were randomized into final treatment groups. A sub-group of animals were then injected with 5 μg recombinant IGF-1 (GroPrep, Adelaide, Australia) for five consecutive days (day 4-8; figure 1A) or injected with vehicle (PBS+BSA). FVB mice were injected intravenously through the tail vein with 5 μg recombinant IGF-1 (total volume of 100 μl) on days 4 through 8 (~24 hours apart) after radiation treatment as previously described [16]. This dose of IGF-1 was chosen because it induces maximal activation of signal transduction pathways in the salivary gland and suppresses radiation-induced loss of salivary gland function [16]. Animals were maintained and treated in accordance with protocols approved by the University of Arizona IACUC.

### Saliva Collection

FVB mice were injected i.p. with carbachol (0.25 mg/kg body weight) as previously described [18] and saliva was collected for 5 min. on days 3, 9, 30, 60, and 90 after radiation treatments. Total saliva was collected by vacuum aspiration from 10-23 mice per treatment group as previously described [23] on ice immediately following carbachol injection into pre-weighed tubes and stored at -80°C. Total proteins present in the collected saliva from 7-8 mice were analyzed by resolving 25 μg total protein on a 10% SDS-PAGE gel, transferred to Immunobilon membrane (Millipore Corporation, Bedford, MA), and immunoblotted with anti-amylase (Sigma-Aldrich, St. Louis, MO). Secondary antibody was conjugated with horseradish peroxidase (anti-HRP conjugated rabbit antibody; Bio-Rad, Hercules, CA) and enhanced chemiluminescence lighting (Pierce Chemical Company, Rockford, IL) was used according to the manufacturer's instructions to detect salivary amylase.

### Histology

Following excision, tissues were immediately fixed in 10% neutral buffered formalin for 24 hours, transferred to 70% ethanol, and embedded in paraffin. Sections of all major salivary glands were cut to 4 μm thickness and processed for standard staining with hematoxylin and eosin by the Histology Service Laboratory in the Department of Cell Biology and Anatomy at the University of Arizona.

### Quantification of acinar cell area

Tissue sections from mice (3-8 mice per group) treated with targeted head and neck radiation were stained for amylase (Sigma-Aldrich, St. Louis, MO) as previously described [24]. Briefly, slides were rehydrated and antigen retrieval performed as described for PCNA. Slides were blocked with 0.5% NEN at room temperature for 1 hour then incubated in anti-amylase primary antibody (1:500) overnight at 4°C. On day 2, slides were washed and incubated in anti-rabbit Cy2-conjugated secondary antibody (1:500) at room temperature for 1 hour, counterstained with DAPI, then mounted with 50% glycerol in 10 mM Tris-HCl. Fluorescent images were visualized on a Leica DM5500 Microscope System and digitally captured with a Spot Pursuit 4 Megapixel CCD camera (Diagnostic Instruments, Inc., Sterling Heights, MI). A figure insert shows a representative image demonstrating the selective staining for acinar cell area. Morphometric analysis was performed with ImagePro 7.0 software (Media Cybernetics, Silver Spring, MD). Positive areas were determined for 25 fields of view (FOV = 0.39 mm^2^) and we were able to obtain a coefficient of variation of <7%, which did not improve with greater numbers of observations per section. Amylase-positive cell area was collected throughout both parotid glands of each mouse on days 30, 60, and 90 following radiation. Data are expressed as the percentage of amylase positive area to the total area of the gland and the threshold fluorescence range was equivalent for all slides imaged.

### Quantification of amylase protein

The collected saliva samples from day 30 were evaluated for total amylase protein using the Bio-Rad Experion System (3-8 mice per group). Samples were loaded into a primed Experion Chip and run using the Experion Software Protein260 Assay. Proteins of different molecular weights were visualized as bands in each sample. Analysis was done from bands located at or around the molecular weight of amylase (54 kDa). Percent total amylase was calculated using the Experion software and graphed for each treatment group.

### Quantification of proliferation

For evaluation of acinar and ductal cell proliferation, unstained tissue sections were processed for anti-PCNA (proliferating cell nuclear antigen, Santa Cruz Biotechnology Inc., Santa Cruz, CA) immunohistochemistry 9, 30, 60, and 90 days after treatment as previously described [18]. Briefly, slides were heated to 37°C for 30 min. and rehydrated in histoclear, graded alcohols, and distilled water washes. Nonspecific peroxidase activity was quenched with 0.3% H_2_O_2_. For antigen retrieval, slides were placed in citrate buffer (pH 6.0) and heated in a microwave oven twice for 5 min, and allowed to cool for 20 min. After washes, the slides were treated according to manufacturer's instructions (Vectastain Elite ABC kit, PK-6101, Vector Laboratories Inc., Burlingame, CA). Color development was achieved with Biogenex DAB incubation for 6 to 8 minutes. Slides were lightly counterstained with Gill's hematoxylin, dehydrated, and mounted in Permount. Tissue sections were observed by standard light microscopy and photomicrographs were taken with a Leica DM5500 with a 4 megapixel Pursuit camera. Positive acinar cells were counted separately from positive ductal cells due to proposed progenitor cells present within the ductal cell network [25]. Individual means for quantification of PCNA positive acinar and ductal cells were determined by averaging the number of positive cells/total number of cells from a minimum of three fields of view/animal (3-5 mice per group; total cells counted ranged from 3,000 to 6,000 per mouse).

### Statistics

Comparison of PCNA and acinar area data, amylase protein content on day 30, as well as normalized saliva flow rates was accomplished by a one-way ANOVA followed by a post-hoc Bonferroni multiple-comparison test. Saliva flow rates were normalized to the respective untreated FVB group for days 3, 30, 60, and 90. Statistical analysis and graphical generation of data were done using GraphPad Prism software (version 5.0, San Diego, CA).

## Results

### Stimulated salivary flow rates are restored in mice receiving post therapy IGF-1

**Figure 2:**
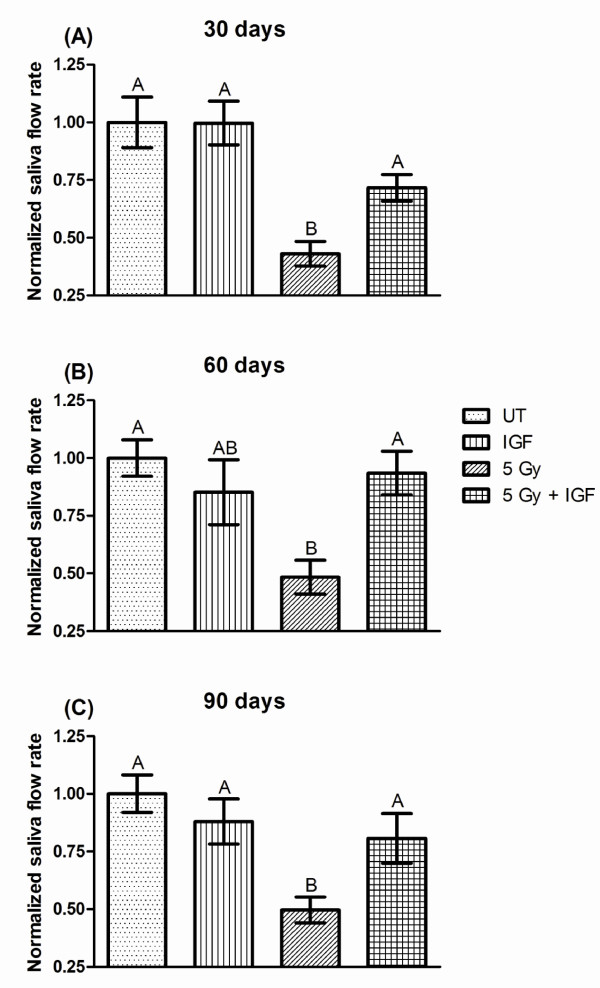
**Salivary gland dysfunction is reversed in mice receiving post therapy IGF-1**. The head and neck region of FVB mice was exposed to a single 5 Gy radiation dose and mice received injections of IGF-1 or vehicle on days 4-8 as depicted in figure 1A. Stimulated salivary flow rates were determined as described in the materials and methods section on days 30 (A), 60 (B), and 90 (C). Irradiated flow rates were normalized to corresponding unirradiated controls after the radiation treatment. The graph represents the mean and standard error of the mean of all data from 10-23 mice per treatment group. Significant differences (p < 0.05) were determined using an ANOVA followed by a post-hoc Bonferroni multiple-comparison test. Treatment groups with the same letters are not significantly different from each other within the same day.

FVB mice exposed to a single radiation dose exhibit significant (*P <*0.05, one-way ANOVA) reductions in salivary function (decreased 43-50%) throughout the time course (figure 2A, B, and 2C) consistent with previously published studies in other animal models [12,26,27]. In contrast, injection with IGF-1 on days 4-8 following radiation increased salivary flow rates to 72% of the untreated control on day 30 compared to irradiated FVB mice (figure 2A). Further increases in salivary flow rates on days 60 and 90 in irradiated mice injected with IGF-1 result in return to untreated flow rates (93 and 81% of untreated controls, figure 2B and 2C). Administration of recombinant IGF-1 to unirradiated FVB mice on days 4-8 did not show significant changes in salivary flow rates at any of the time points evaluated (85-100% of untreated controls) indicating that IGF-1 does not affect glandular function in unstressed environments. These functional data suggest that post therapy IGF-1 can alter the response of salivary glands to radiation damage leading to restoration of function.

### Post therapy IGF-1 restores functional acinar cell area and amylase secretion in irradiated mice

**Figure 3:**
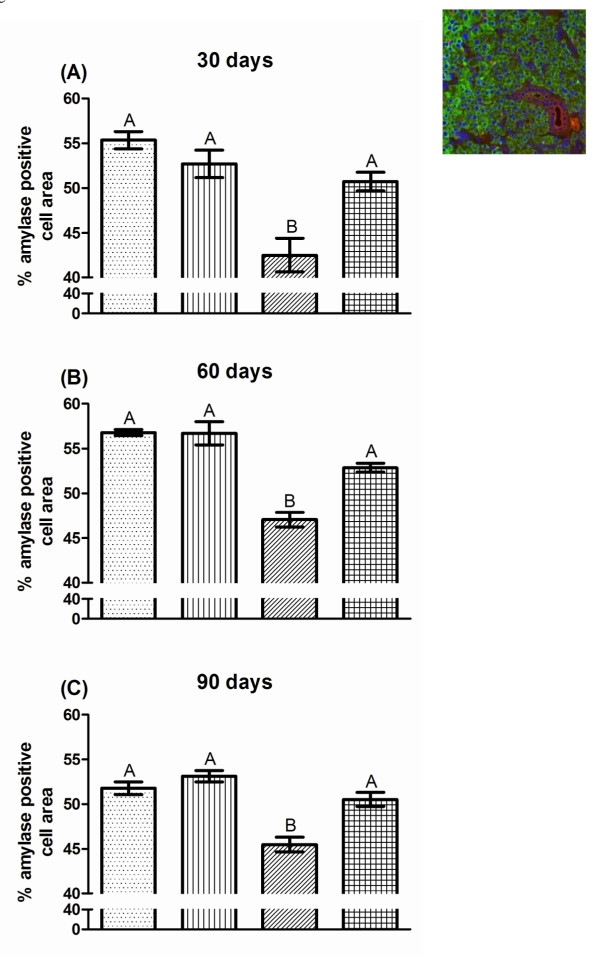
**Post therapy IGF-1 restores functional acinar cell area in irradiated mice**. The head and neck region of FVB mice was exposed to a single 5 Gy radiation dose and mice received injections of IGF-1 or vehicle on days 4-8 as depicted in figure 1A. Parotid glands were removed on days 30 (A), 60 (B), and 90 (C) after the radiation treatment. Tissues were embedded into paraffin and immunohistochemistry was performed according to the materials and methods section. The insert is a representative image with amylase-positive acinar cells (green), cytokeratin-positive ductal cells (red), and cell nuclei (blue). The graph represents the percentage area of amylase-positive cells in the field of view. All data are composed of the mean and standard error of the mean from 3-8 mice per group. Significant differences (p < 0.05) were determined using an ANOVA followed by a post-hoc Bonferroni multiple comparison test. Treatment groups with the same letters are not significantly different from each other within the same day.

**Figure 4:**
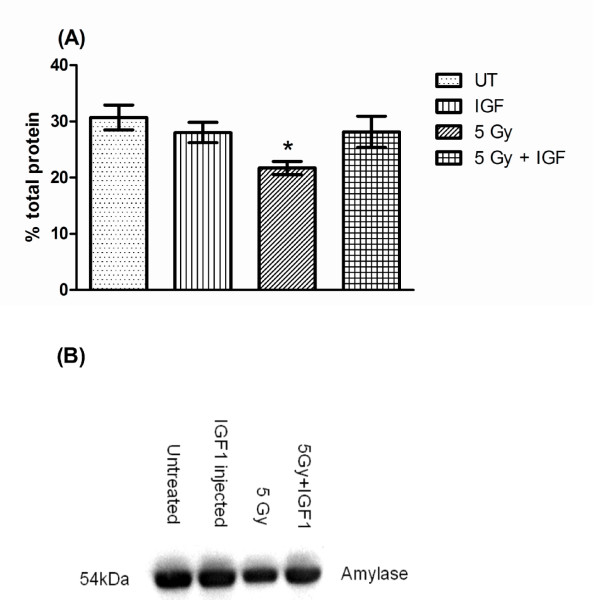
**Post therapy IGF-1 restores amylase content in stimulated saliva 30 days following radiation**. The head and neck region of FVB mice was exposed to a single 5 Gy radiation dose and mice received injections of IGF-1 or vehicle on days 4-8 as depicted in figure 1A. In (A), stimulated saliva samples from figure 2A were collected and analyzed for total protein content as described in the materials and methods section. The graph represents the percentage of amylase protein (ranging from 50-57 kD). All data are composed of the mean and standard error of the mean from 7-8 mice per group. Significant differences (p < 0.05) were determined using an ANOVA followed by a Bonferroni test. (*) in figure 4A indicates significant differences between irradiated mice receiving post-therapy IGF-1 or vehicle. A representative Western blot as described in the materials and methods section of day 30 saliva samples is shown in (B).

On all days, a consistent 14-24% reduction in amylase-positive cell area is measured for FVB mice irradiated with a single 5 Gy dose that received vehicle injections. This reduction was significantly different from untreated FVB, IGF-1 injected, and irradiated mice receiving post therapy IGF-1 (*P <*0.05, one-way ANOVA). The acinar cell areas for untreated FVB and IGF-1 injected mice are not significantly different at any time point. Post therapy injections of IGF-1 on days 4-8 after irradiation lead to a significant increase in amylase-positive acinar cell area which is not significantly different from both unirradiated groups (93-98% of untreated controls, figure 3). In addition, evaluation of the total amylase protein content in stimulated saliva on day 30 shows that restoration of acinar cell areas in IGF-1 injected mice indeed increase amylase content in stimulated saliva (90% of untreated controls, figure 4). In contrast, irradiated mice secrete less amylase (decreased 29%) compared to unirradiated control saliva samples. The increase in amylase-positive cell area and amount of amylase protein in stimulated saliva on day 30 (figure 4) in irradiated mice receiving IGF-1 correlates with increases in stimulated salivary flow rates on the corresponding time points (figure 2A, B, and 2C).

### Proliferation indices return to untreated levels in irradiated mice receiving post-therapy IGF-1

**Figure 5:**
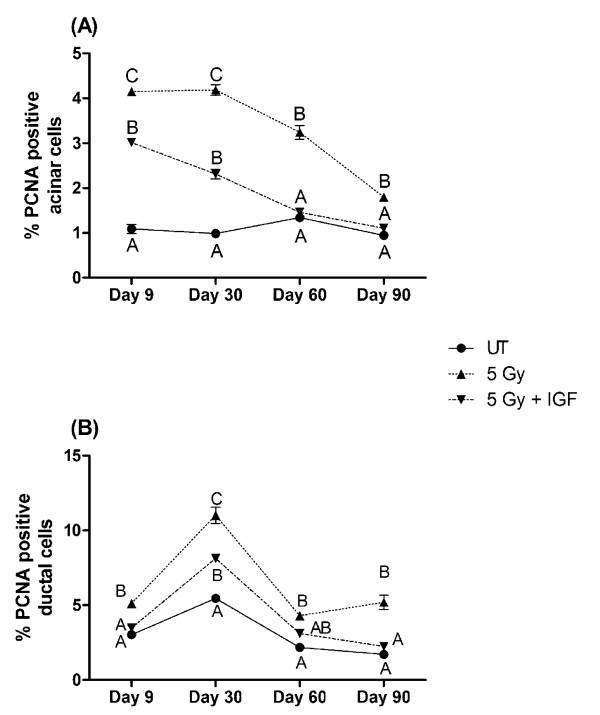
**Proliferation of parotid acinar and ductal cells following radiation (single 5 Gy dose) in FVB or IGF-1 injected mice**. The head and neck region of FVB mice was exposed to a single 5 Gy radiation dose and mice received injections of IGF-1 or vehicle on days 4-8 as depicted in figure 1A. Parotid glands were removed 9, 30, 60, and 90 days after the radiation treatment. Tissues were embedded into paraffin and immunohistochemistry was performed using an antibody against PCNA. The graph represents the number of acinar cells with positive PCNA staining as a percentage of the total number of acinar (A) or ductal (B) cells in the field of view. The data is displayed as the mean and the standard error of the mean of all data from 3-8 mice per group. Significant differences (p < 0.05) were determined using an ANOVA followed by a post-hoc Bonferroni multiple-comparison test. Treatment groups with the same letters are not significantly different from each other within the same time point.

FVB mice exposed to 5 Gy have a 4-fold increase in PCNA positive salivary acinar cells at days 9 and 30 when compared to untreated controls (*P <*0.05, one-way ANOVA, figure 5A). At days 60 and 90, irradiated FVB mice continue to show elevated PCNA-positive acinar cell levels that is statistically higher than untreated controls (*P <*0.05, one-way ANOVA). In contrast, the number of PCNA positive acinar cells in irradiated mice injected with IGF-1 gradually decreases from elevated levels (2-3 fold) on days 9 and 30 to levels similar to untreated mice on days 60 and 90 (figure 5A). At later time points (days 60-90), the percentage of PCNA positive acinar cells in mice receiving post-therapy IGF-1 are not statistically different from untreated controls (*P <*0.05, one-way ANOVA). In irradiated mice, the level of PCNA positive ductal cells remains significantly elevated throughout the observation period similar to the profile detected in acinar cells (*P <*0.05, one-way ANOVA), figure 5B). Interestingly, the level of PCNA positive ductal cells in irradiated mice receiving IGF-1 only differs from untreated control mice on day 30. This profile within the ductal cells of irradiated IGF-1 injected mice is strikingly different from the profile of the acinar cells which demonstrate a gradual decrease in PCNA (compare 5A and B).

## Discussion

Radiation treatment of head and neck cancer patients causes significant chronic damage to the salivary glands and there are few therapeutic options available to improve the patients' quality of life. The current findings indicate that post therapy injections of IGF-1 following radiation restore salivary gland function (figure 2), which poses several clinical advantages. Implications include the possibility of restoring salivary gland function in specific patients by delivering an IGF1-type compound to restore deficient salivary gland function, even after radiation therapy.

The reduction in stimulated salivary flow rates for animals receiving a single radiation dose of 5 Gy is significant throughout the 90 day observation period (figure 2). These findings are in agreement with previous studies using single doses of radiation (1-5Gy) [16,19]. Post therapy administration of IGF-1 on days 4-8 after radiation exposure significantly increases salivary flow rates at all time points. On days 60 and 90 flow rates have been restored to untreated levels (figure 2). This further establishes a critical role for IGF-1 in the signaling pathways that manage the cellular response to radiation in the salivary glands. It also suggests that post therapy activation of specific downstream molecules may be an effective targeted therapy for the restoration of salivary gland function.

Loss of acinar cells in response to radiation has been widely discussed. While significant levels of apoptosis immediately following radiation have been observed in mice [16,18-20], the presence of apoptosis in irradiated rats has been more variable [28-30]. Because acinar cells contribute a majority of saliva constituents including amylase and water, loss of these cells would impair normal saliva composition and lead to reduction in saliva flow. Our results indicate that reduction of stimulated salivary flow rates as a physiological parameter is closely related to reductions in amylase-positive acinar cell area in parotid glands (figure 3). The percentage of amylase positive cells is a useful indicator for the evaluation of actively secreting parotid acinar cells [9]. Our findings further indicate that the loss of amylase positive cells is correlated with a decrease in amylase protein secretion (figure 4). Post therapy administration of IGF-1 to irradiated mice results in significant increases of amylase-positive cells over the course of the observation period compared to irradiated animals that received vehicle alone (figure 3). While irradiated animals have consistently reduced areas of amylase-positive cells on days 30, 60, and 90, administration of IGF-1 after radiation results in increased amylase-positive cells and improved amylase secretion indicative of a restoration of functioning acinar cells (figures 3 and 4). A common observation in salivary glands exposed to radiation is atrophy and loss of glandular weight indicative of cell loss (reviewed in [17]), and our results implicate that functional acinar cell area is restored in animals receiving post therapy IGF-1 injections.

An interesting finding of our study is the influence of post therapy IGF-1 administration on PCNA, a marker of cell proliferation. Tissue homeostasis is maintained by keeping a balance between cell growth and cell death. Salivary glands, unlike other radiosensitive tissues, consist of highly differentiated cells that proliferate slowly (reviewed in (24)). In our study, we observe increased levels of PCNA positive acinar cells in animals receiving a single dose of 5 Gy (figure 5) which could be indicative of compensatory proliferation [31]. In rapidly cycling tissues, compensatory proliferation is a mechanism to replace lost cells; however in slowly cycling tissues, compensatory proliferation correlates with loss of function [31-33]. Our study concurs with these studies in that radiation-induced compensatory proliferation in the salivary gland correlates with reduced physiologic function. It is feasible that the cells actively cycling in the salivary gland have an undifferentiated phenotype that does not produce an appreciable amount of saliva. While the level of PCNA positive acinar cells remains significantly elevated in irradiated animals, mice receiving post therapy IGF-1 decreased the levels of PCNA positive acinar cells to back to control levels on days 60 and 90 (figure 5A). Our study corroborates radiation-induced compensatory proliferation that has been reported in the submandibular salivary glands of mice [34,35] and the parotid salivary glands of rats [36]. Interestingly, administration of pilocarpine prior to radiation treatment of rats resulted in increased compensatory proliferation and preservation of salivary function [36,37]. One explanation for the difference between our study and the pilocarpine study is the timing of administration. Our study evaluated alterations in proliferation indices using a post-therapy model (figure 1A) whereas pilocarpine was administered prior to radiation treatment [36].

Currently the salivary gland stem cell is unknown; however, there is growing evidence that progenitor cells are present within the ductal cell network. Immunohistochemical staining of submandibular salivary glands for the stem cell marker c-kit revealed its localization to the excretory duct [25]. In addition, transplantation of salivary cells enriched for c-kit into irradiated mouse salivary glands restores salivary gland function [25]. Our results in irradiated mice demonstrate an increase in PCNA positive ductal cells across the entire time course similar to the acinar cells (figure 5). In contrast, mice receiving post therapy IGF-1 have an increase in PCNA positive ductal cells only at the day 30 time point after radiation treatment. It is possible that IGF-1 may transiently influence the stem/progenitor population within the ductal network in order to restore salivary gland function; however more studies are required to test this hypothesis.

One potential concern for the use of growth factors such as IGF-1 for clinical applications is the effect as a tumor promoter. However, salivary gland tumors were not detected in any of the animals that received five consecutive injections of IGF-1 in our studies. Furthermore, it is more likely that drugable analogs of IGF-1 specific to the tissue or pathways involved would be developed and administered clinically. In addition, the salivary glands could be specifically treated through cannulation of the salivary duct. The long-term translation of this study involves the identification of these pathway specific molecules that could be utilized to specifically target and restore salivary gland function following clinical radiation therapy.

## Conclusions

The results of our study provide further support for the involvement of IGF-1 or analogous compounds in the preservation and restoration of salivary glands following radiation which can lead to diminished side effects and provide head and neck cancer patients with the possibility for improved quality of life.

## Abbreviations

(IGF-1): Insulin-like growth factor-1; (PCNA): proliferating cell nuclear antigen.

## Competing interests

OG, JLF, KV, and RB have nothing to declare. KHL is listed as an inventor on U.S. Patent Application No. 12/304,359.

## Authors' contributions

OG participated in the study design, carried out physiology experiments, conducted statistical analysis on all data, and drafted the manuscript. JLF contributed to the physiology experiments, and completed all amylase analysis experiments. KV completed the proliferation analysis. KHL and RB conceived the studies and participated in design of the experiments. KHL oversaw and coordinated the studies and finalized writing of the manuscript. All authors read and approved the final manuscript.

## Pre-publication history

The pre-publication history for this paper can be accessed here:

http://www.biomedcentral.com/1471-2407/10/417/prepub
